# *In vitro* beta-cell killing models using immune cells and human pluripotent stem cell-derived islets: Challenges and opportunities

**DOI:** 10.3389/fendo.2022.1076683

**Published:** 2023-01-16

**Authors:** Clémentine Halliez, Hazem Ibrahim, Timo Otonkoski, Roberto Mallone

**Affiliations:** ^1^ Université Paris Cité, Institut Cochin, CNRS, INSERM, Paris, France; ^2^ Assistance Publique Hôpitaux de Paris, Service de Diabétologie et Immunologie Clinique, Cochin Hospital, Paris, France; ^3^ Stem Cells and Metabolism Research Program, Faculty of Medicine, University of Helsinki, Helsinki, Finland; ^4^ Department of Pediatrics, Helsinki University Hospital, Helsinki, Finland

**Keywords:** beta-cells, T-cells, cytotoxicity, type 1 diabetes, iPSCs, stem-cell-derived islets, human leukocyte antigen (HLA)

## Abstract

Type 1 diabetes (T1D) is a disease of both autoimmunity and β-cells. The β-cells play an active role in their own demise by mounting defense mechanisms that are insufficient at best, and that can become even deleterious in the long term. This complex crosstalk is important to understanding the physiological defense mechanisms at play in healthy conditions, their alterations in the T1D setting, and therapeutic agents that may boost such mechanisms. Robust protocols to develop stem-cell-derived islets (SC-islets) from human pluripotent stem cells (hPSCs), and islet-reactive cytotoxic CD8^+^ T-cells from peripheral blood mononuclear cells offer unprecedented opportunities to study this crosstalk. Challenges to develop *in vitro* β-cell killing models include the cluster morphology of SC-islets, the relatively weak cytotoxicity of most autoimmune T-cells and the variable behavior of *in vitro* expanded CD8^+^ T-cells. These challenges may however be highly rewarding in light of the opportunities offered by such models. Herein, we discuss these opportunities including: the β-cell/immune crosstalk in an islet microenvironment; the features that make β-cells more sensitive to autoimmunity; therapeutic agents that may modulate β-cell vulnerability; and the possibility to perform analyses in an autologous setting, i.e., by generating T-cell effectors and SC-islets from the same donor.

## Introduction

Type 1 diabetes (T1D) is an autoimmune disorder causing pancreatic β-cell loss that results in progressive failure of insulin secretion to control blood glucose levels. There are around 537 million diabetic patients worldwide, T1D representing 5-10% of these cases (1). T1D incidence has drastically increased, especially in young children, with an annual increase of 3-4% over the last 3 decades. T1D is characterized by an autoimmune attack on β-cells. Abnormal interactions between β-cells and immune cells include autoantibody (AAb) production by B-cells and the engagement of cytotoxic CD8^+^ T-cells as the final actors of β-cell destruction (2–6).

**Figure 1:**
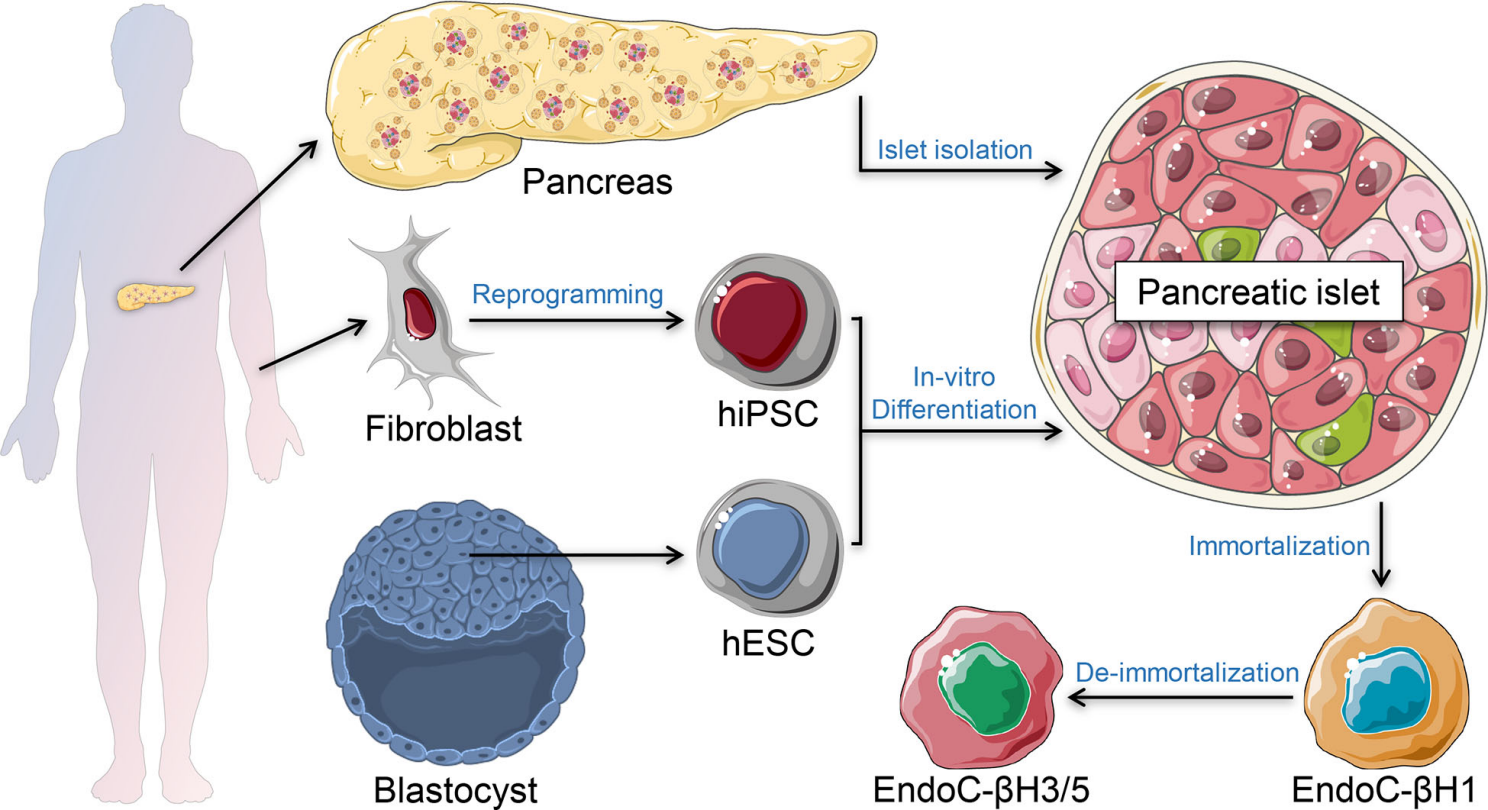
β-cell models. Primary human islets can be isolated *ex vivo* from human pancreatic tissue of brain-dead organ donors. Stem cell-derived islets can be generated *in vitro* using human induced pluripotent stem cells (hiPSC) or human embryonic stem cells (hESC). Immortalized β-cell models are generated from human insulinomas propagated *in vitro.* De-immortalized β-cell-lines are generated to enhance functional responses, mimicking adult primary β-cells.

The genetics of T1D is complex. It is a polygenic disease, with more than 60 risk loci identified that account for ~80% of T1D heritability (7). Many of these genes are expressed in human islet cells and immune cells with known functional roles, suggesting that T1D pathogenesis involves both cell types (8–10). While T1D prevalence in the general population is ~0.1%, family and twin studies suggested concordance rates of around 50% and 7% for identical twins and siblings, respectively (11–13). Around 50% of T1D genetic risk is related to the human leukocyte antigen (HLA) region, especially in the loci of HLA class II DR and DQ (14). These loci and the other genetic variants associated with T1D, whether coding or non-coding, require experimental validation of their impact on β-cell destruction to recapitulate and understand the pathophysiology of T1D. Since obtaining pancreatic tissue from live T1D patients is a rare occurrence (15), several β-cell models have been developed to recapitulate and investigate the progression of T1D. The most common *in vivo* autoimmune models for studying T1D are the non-obese diabetic (NOD) mouse and the BioBreeding (BB) rat (16, 17). Many T1D pathogenetic elements and immune pathways were identified using these valuable animal models. However, the physiology of β-cell development and function as well as the pathophysiology of T1D in humans are fundamentally different compared to rodents (18–20). Therefore, several human β-cell models were established to understand and recapitulate the pathophysiology of diabetes (**Figure 1**). Furthermore, understanding the crosstalk between the β-cells and the immune cells is crucial to generate immune-protected β-cells for cell replacement therapy. In this review, we summarize recent progress in T1D disease modeling and future opportunities using *in vitro* systems of β-cell and T-cell crosstalk.

## β-cell models

### Primary human islets

The most biologically relevant model to study human islet cells are the primary human islets isolated from brain-dead organ donors. Scientists have been optimizing their isolation since the early 70s (21), providing increasingly higher success rates, yields and purity (22–24). However, there are many variables that affect the isolation outcome, such as the age and sex of the donor, the duration of enzymatic digestion and the enzyme lot (25). These and other factors are reflected by the variable reproducibility of experiments using different preparations (26). Relevant to many investigations, the *in vitro* addition of stressors such as pro-inflammatory cytokines may often elicit milder effects in primary islets than in other β-cell models, e.g. on HLA Class I upregulation (27), which likely reflects peri-mortem and tissue isolation stress conditions (28). Moreover, once isolated, human islets need to be utilized within a brief period (~7 days) due to their progressive death *in vitro*. To extend the time of culture and to minimize the variability in preparation purity, a hanging-drop-based re-aggregation process of dispersed primary human islets was developed, enriching for endocrine cells, and extending the culture duration for up to 4 weeks (29). These human pseudo-islet microtissues are marketed by InSphero as 3D InSight™. Nonetheless, another major drawback of primary human islets is their limited and often unpredictable availability, which relies on organ donors and long, expensive and highly specialized isolation procedures.

### EndoC-βH cell-lines

**Figure 2:**
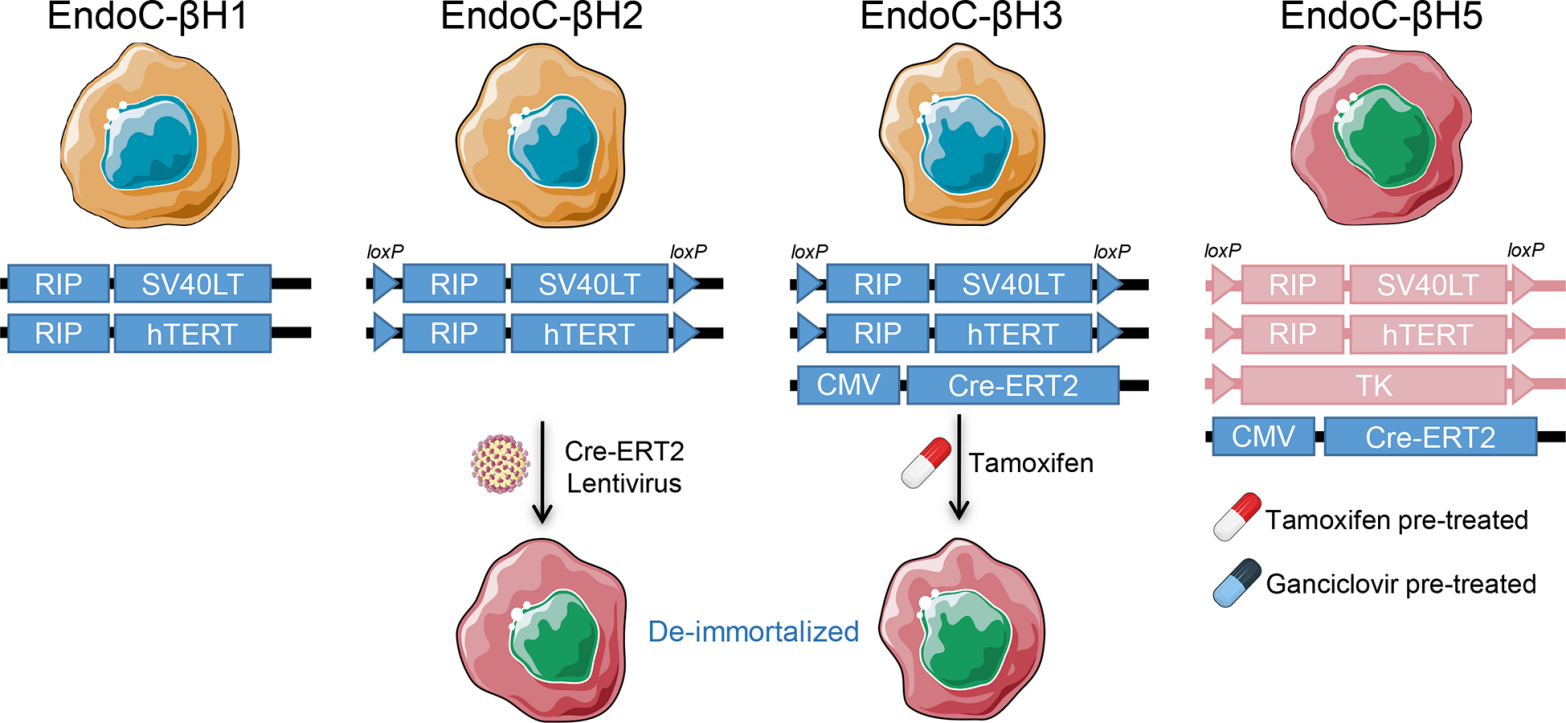
EndoC-βH lines. The human insulinoma EndoC-βH lines and their respective immortalization and de-immortalization constructs. RIP, Rat insulin promoter; Cre-ERT2, Tamoxifen-regulated Cre recombinase and estrogen receptor T2; TK, Thymidine kinase.

One alternative to bypass the limitations of human primary islets is to generate immortalized human β-cell lines that can be expanded *in vitro.* Several cell lines have been generated using diverse techniques, but most of them failed to show proper β-cell phenotype and glucose-stimulated insulin secretion (30). These include the βLox5 line, which was generated from purified adult pancreatic β cells transformed using a retroviral vector encoding human telomerase reverse transcriptase (hTERT), SV40LT antigen, and rasval12 oncogene (31). Although widely used, their insulin secretion remains substantially lower than in human islets. A major advance has been provided by the EndoC-βH cells developed by Scharfmann et al. (32), which have become the most commonly used lines (**Figure 2**). The first of these lines, called EndoC-βH1, was generated from human fetal pancreatic tissue sequentially transduced with lentiviruses expressing the SV40LT oncogene and hTERT, both under the control of a rat insulin promoter. The transduced cells were implanted under the kidney capsule of severe combined immunodeficient (SCID) mice to form insulinomas, followed by their retrieval and *in vitro* expansion. Due to the proliferative capability of these cells, their insulin content and secretion were modest. A second-generation cell line (EndoC-βH2) was therefore obtained with a conditional immortalization cassette flanked by LoxP sites that can be excised upon Cre recombinase lentiviral transduction to de-immortalize the cells. This de-immortalization step leads to a more mature β-cell phenotype and enhanced insulin content and secretion (33). To avoid the need for Cre transduction, the EndoC-βH2 cells were then lentivirally transduced to stably express the tamoxifen inducible Cre recombinase (Cre-ERT2), generating the EndoC-βH3 cell-line (34). The latest version recently developed called EndoC-βH5 was obtained by a tamoxifen excisable construct which is similar to EndoC-βH3, but contains a thymidine kinase expression cassette. This suicide gene allows the elimination of residual SV40LT/hTERT-expressing cells using the drug ganciclovir. Due to this selection, the cells lose their proliferative capacity and cannot be propagated in culture. However, they display a physiological 6-fold glucose-stimulated insulin secretion and improved potentiation by other secretagogues, e.g., glucagon-like peptide-1 and gastric inhibitory polypeptide, mimicking primary β-cells (35). This cell line is provided by Human Cell Design as single-use vials. Although EndoC-βH lines carry some chromosomal aberrations and do not recapitulate the biology of other islet endocrine cells, they are an indispensable model for studying β-cells (36). They can also be genetically manipulated using chemical transfection or electroporation with plasmids or small interfering RNAs (siRNAs) (36). Others have succeeded in creating knock-in and knock-out models by lentiviral transduction of EndoC-βH1 (37, 38). However, it remains difficult to obtain genetically edited monoclonal cell lines due to their low proliferation rate.

Another ECN90 β-cell line was more recently developed by the Scharfmann’s team from neonatal pancreas using a similar protocol (39). This line presents the advantage to express the most prevalent HLA Class I allele HLA-A2 and can therefore be more conveniently used for CD8^+^ T-cell cytotoxicity assays (3) and for the identification of HLA-A2-bound antigenic peptides (27).

### Stem-cell-derived islets (SC-islets)

An alternative model combining many positive features of primary human islets and the EndoC-βH lines is provided by stem-cell-derived islets (SC-islets). SC-islets can be generated from human pluripotent stem cells (hPSCs), either utilizing human embryonic stem cells (hESCs, where available) or human induced pluripotent stem cells (hiPSCs). hPSCs have two properties making them a valuable system to generate pancreatic islets *in vitro*. First, they have the ability to self-renew and proliferate indefinitely; second, they can differentiate into specialized cell types of the three germ layers. The first protocol to induce insulin-producing cells from hESCs was by spontaneous differentiation (40). This potential emphasized how directing the differentiation to generate SC-islets may provide a powerful tool. By using different growth factors and small molecules to mimic the signalling cues discovered by studying the development of the mouse pancreas, it was possible to generate SC-islets *in vitro* (41, 42). Many protocols were developed for stepwise differentiation of hPSCs to generate SC-islets (43–50). Although the generated SC-islets were considered functionally inferior compared to human primary islets, recent advancements in differentiation protocols showed major improvements, yielding differentiated SC-islets that closely mimic native human islets from a functional and transcriptional standpoint (50, 51). Despite the different protocols used across different labs, many succeeded in generating functional SC-islets from both hESCs and hiPSCs within a culture time of ~35 days, with some differences in cytoarchitecture and functional capacity. The variability reported in generating derivatives from different iPSCs including SC-islets is mainly due to the different genetic background of the donors (52, 53). The generated SC-islets comprise the 3 major cell types of pancreatic endocrine cells (β-, α- and δ-cells), in addition to a subpopulation of enterochromaffin (EC)*-*like cells (46, 50). However, the number of EC-like cells decreases upon further maturation *in vitro* (50). The presence of the major pancreatic endocrine cell types in SC-islets mimics the complexity of isolated primary human islets. Since the role of α-cells in T1D is gaining momentum (54), their presence in SC-islets paves the way to study why they become dysfunctional yet not destroyed in T1D (55), and their impact on β-cell dysfunction and immunogenicity. The use of fluorescent reporters under the control of α-, β- and δ-specific promoters may moreover allow to distinguish endocrine lineages (56, 57). It is important to note that SC-islets lack other cells and structural molecules that are present within the islet niche microenvironment *in vivo*, including endothelial, mesenchymal, immune and neural cells along with their associated extracellular matrix.

Once the SC-islets are generated, they are easily maintained in 3D suspension culture, though not indefinitely, limiting their on-demand availability compared to immortalized β-cell lines. The cell yields of SC-islets are also lower than what can be obtained with immortalized β-cell lines. Increasing the yields of SC-islets is feasible but cost-demanding. Recently, a state-of-the-art method for long-term islet cryopreservation succeeded in recovering SC-islets with high viability and functionality even after prolonged cryopreservation (58). This may allow the generation of high-quality SC-islets on a large scale and their storage until required for further experimentation or β-cell replacement therapy.

SC-islets provide an outstanding model for studying β-cell development and function, and for drug screening. Since SC-islets originate from stem cells, they can be genetically modified by introducing specific mutations into wild-type hESCs or hiPSCs, as well as correcting mutations or disease-associated gene variants in hiPSCs generated from diabetic patients. A plethora of diabetes-associated genes have already been modelled by combining the use of SC-islets and CRISPR genome editing (59–73). Even for a complex disease like T1D, the possibility to model the effect of T1D risk and protective genes or single nucleotide polymorphisms on β-cell development and T-cell-mediated cytotoxicity has become feasible (74). Although SC-islets from T1D-donor-generated iPSCs were reported to be functionally similar compared to their counterparts from healthy donors (75, 76), further investigation is needed on more donors with more in-depth characterization of the phenotype to confirm these findings.

**Figure 3:**
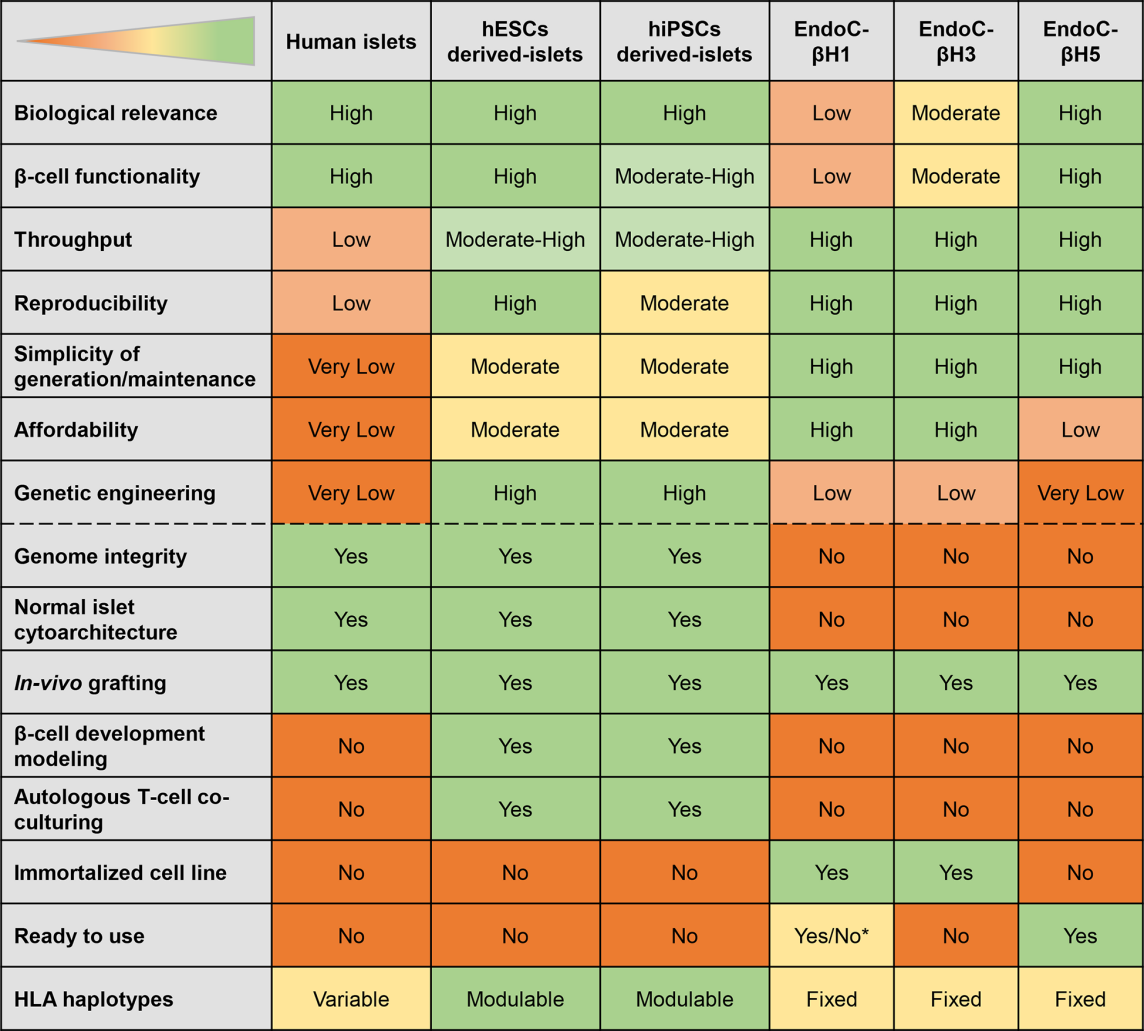
Features of different β-cell models. The table displays the strengths (green) and weaknesses (orange) of different models. *EndoC-βH1 ready to use depending on the application envisaged.

Based on their properties, SC-islets can be considered to have the utmost potential for modelling T1D (**Figure 3**). The mentioned β-cell models provide the target cells that can be used in combination with effector CD8^+^ T-cells to mimic and understand the pathophysiology of T1D. Relevant to these studies, β-cell lines come with fixed HLA haplotypes reflecting that of the cell donor, while these haplotypes can be modulated for SC-islets by using different donors. The next section discusses the approaches to generate T-cell models that can be utilized for targeting β-cells.

## Islet-reactive CD8^+^ T-cell models

**Figure 4:**
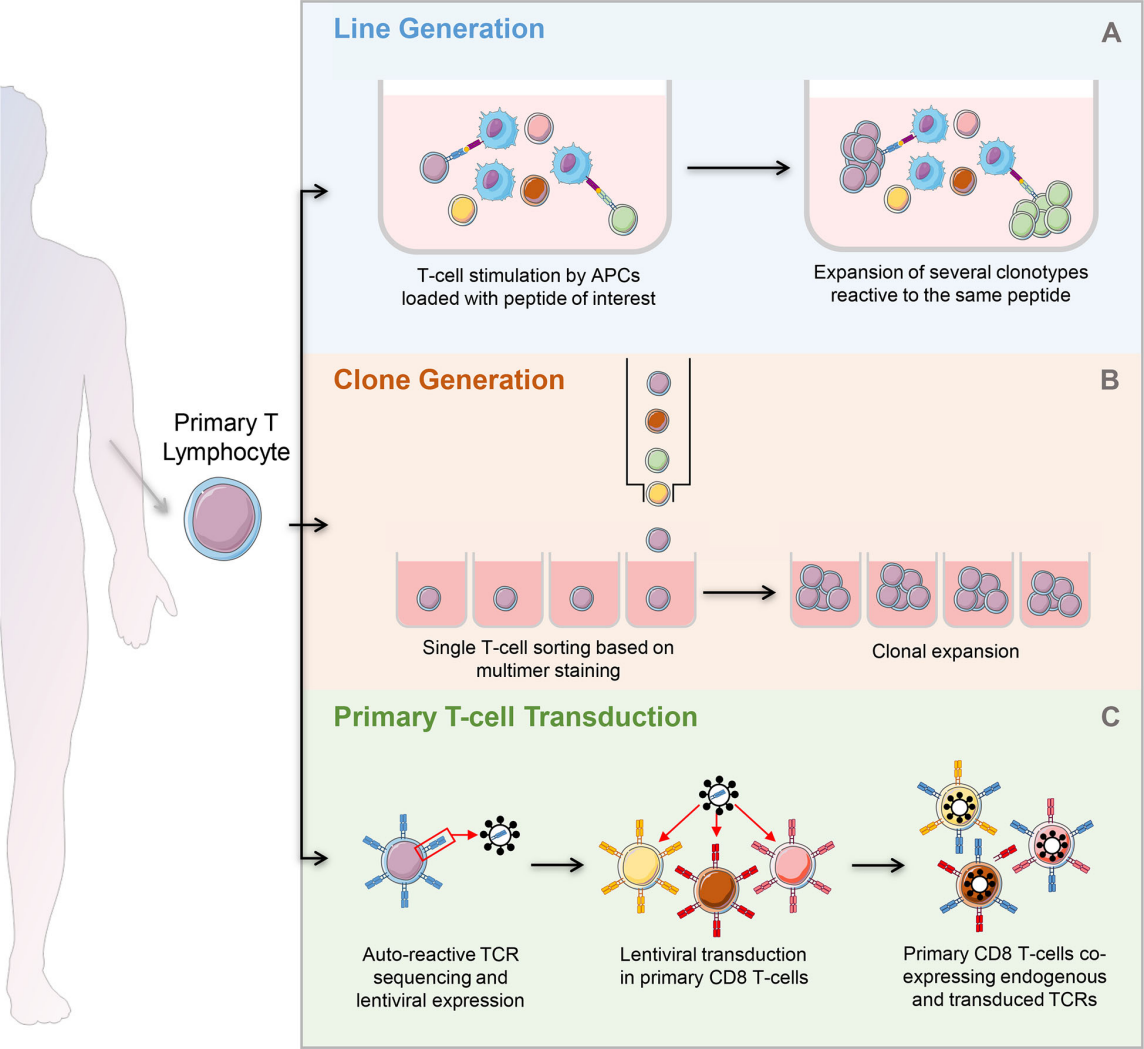
Strategies to generate autoreactive T-cells. **(A)** Generation of T-cell lines: after magnetic selection from PBMCs, T-cells are stimulated by APCs loaded with the peptide of interest. Peptide specific T-cells undergo clonal expansion, leading to the development of several clonotypes reactive to the same peptide. **(B)** Clone generation: T-cells are isolated and used directly *ex vivo* or after a short stimulation with APCs loaded with the peptide of interest. They are single-sorted based on multimer staining and plated for further expansion. Each well will contain a single clonotype. **(C)** Primary T-cell transduction: after magnetic selection from PBMCs, primary T-cells are lentivirally transduced to express an islet-reactive TCR. The primary T-cell transductants obtained co-express their endogenous TCR and the islet-reactive TCR of interest. PBMCs, peripheral blood mononuclear cells; APCs, antigen presenting cells.

**Figure 5:**
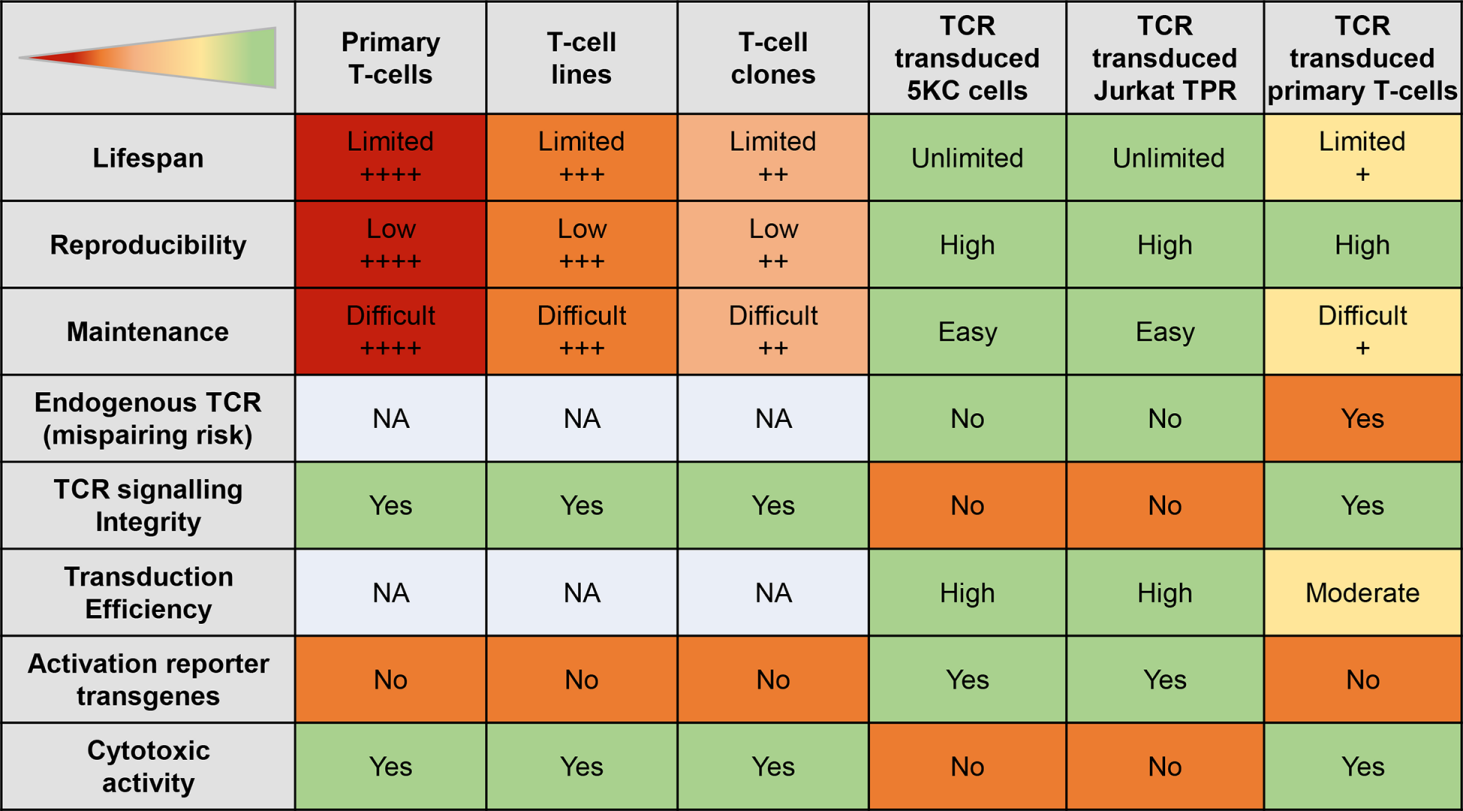
Features of different autoreactive T-cell models. The table displays the strengths (green) and weaknesses (red) of different T-cell models. The + signs indicate the level of the status; NA, not applicable.

Abnormal activation of many types of antigen-presenting cells (APCs) plays a role in the pathophysiology of T1D (77). However, T-cells, particularly CD8^+^ T-cells, are the final effectors of pancreatic β-cell destruction. They are also the most abundant immune subset infiltrating the islets of T1D patients (78). Different strategies are available to generate islet-reactive T-cells (**Figure 4**), and their strengths and weaknesses are summarized in **Figure 5**.

The most obvious way to study these lymphocytes and their interaction with β-cells would be to isolate them from peripheral blood mononuclear cells (PBMCs). For instance, total CD8^+^ T-cells can be isolated from PBMCs by magnetic selection, and those reactive to islet antigens can be sorted after labelling using HLA Class I multimers (79). When directly studied *ex vivo*, these lymphocytes have a high clinical relevance and reliable recognition of their cognate peptide. However, they are found at low frequencies (1-50/10^6^ CD8^+^ T-cells for a given antigenic peptide) in the circulation of T1D and healthy donors, which corresponds to the expected range for any naïve CD8^+^ T-cell specificity (80). Although higher frequencies have been reported by other teams (3, 81–86), in our hands this range displays limited variation across antigenic specificities and individuals, in line with the largely naïve phenotype of these circulating islet-reactive CD8^+^ T-cells. In comparison, CD8^+^ T-cells directed against peptides derived from commonly encountered viruses, e.g., Influenza virus, EBV, CMV are 10- to 1,000-fold more frequent (3, 82, 86). Furthermore, we have recently shown that these islet-reactive T-cells do not display higher frequencies in the peripheral blood of T1D patients than in healthy subjects (3, 27, 82). Because of this low frequency and, subsequently, low isolation efficiency, they cannot be used directly *ex vivo* for high-throughput β-cell/CD8^+^ T-cell interaction models.

The first solution to reach higher cell numbers is to generate T-cell lines (i.e., polyclonal, comprising multiple different T-cell clonotypes recognizing the same antigens, often admixed with irrelevant T-cells) or clones (i.e. monoclonal, comprising a single T-cell clonotype). To obtain T-cell lines, islet-reactive lymphocytes are labelled with HLA Class I multimers or activation markers following a short *in vitro* antigen stimulation and single-sorted in culture plates for further expansion. Single-cell sorting can be done directly *ex vivo* or following stimulation with peptide-loaded APCs (e.g., monocyte-derived dendritic cells or unfractionated PBMCs) in order to obtain a preliminary antigen-driven expansion, usually boosted through the addition of cytokines such as IL-2, IL-7, IL-15. In both cases, single cells are plated in individual wells and expanded, so that all the progeny represents a single clonotype. The most studied islet-reactive CD8^+^ T-cell clone is 1E6, which recognizes a preproinsulin (PPI)_15-24_ peptide (87, 88). The generation of both T-cell lines and clones is labour-intensive, while their length of *in vitro* expansion is limited, as these cells need to be periodically (every 10-14 days) restimulated by peptide-pulsed APCs or mitogens and eventually undergo exhaustion and/or functional anergy. Moreover, this expansion requires the addition of third-party PBMCs that act as feeders to sustain growth. Although these feeder cells are routinely irradiated to avoid their expansion, they can occasionally survive in culture and dilute the peptide-reactive T-cell fraction of interest. Moreover, only a small fraction of clones (<10% in our hands) can be proficiently expanded *in vitro* and confirm the antigen reactivity against which they were originally selected. Another limitation of clones obtained from primary CD8^+^ T-cells is their variable behaviour across different stimulation cycles and limited *in vitro* expansion, limiting the number of single-batch cells available for high-throughput assay platforms.

In order to overcome the limited expansion and variable reproducibility of clones, immortalized “avatars” of autoreactive T-cells have been developed by transducing T-cell receptors (TCRs) directed against β-cell peptides into different host cells. Hybridomas provide a first type of recipient cells. They are obtained by immortalizing a T-cell through fusion with a tumour cell line. The widely used 5KC line is a murine hybridoma obtained from a mouse CD4^+^ T-cell fused with thymoma cells (89–91). It constitutively expresses murine CD4, but can be transduced with human CD4 or CD8, and CD3 molecules in order to increase their responsiveness to cognate peptide following introduction of a human TCR (92). A modified version elaborated in 1993 does not express an endogenous TCR, reducing the risk of mispairing when an exogenous TCR is transduced (91).

Another interesting model is the Jurkat cell line. It is an immortalized human CD4^+^ T-cell line obtained from a non-Hodgkin lymphoma patient (93). A derived cell line developed by the Steinberger Lab, called Jurkat Triple Parameter TCR signalling reporter (TPR), presents several advantages. First, it is knocked-out for endogenous TCRα/β subunits, thus decreasing the risk of mispairing between the endogenous and exogenous TCR. Second, it expresses the CD8αβ co-receptor to stabilise TCR-HLA interactions, as well as three fluorescent reporter genes under the transcriptional control of nuclear factor of activated T-cells (NFAT), NF-κB and activator protein (AP)-1. These three transcription factors are activated upon TCR triggering, thus leading to fluorescent protein expression when peptide-HLA recognition takes place. These fluorescent reporters offer a simple read-out of T-cell activation (94). Furthermore, Jurkat cells present a TCR transduction efficiency close to 100% (94).

Both 5KC and Jurkat have the advantage to be immortalized cell lines, easy to grow in a highly reproducible fashion. However, they are derived from CD4^+^ T-cells and therefore lack cytotoxic machinery. Moreover, being derived from highly mutated tumour cells, they show abnormalities in TCR signalling pathways (95), endowing them with a lower responsiveness to their cognate peptide compared to primary CD8^+^ T-cell lines and clones. If we consider the fact that autoimmune TCRs have generally lower affinity than those recognizing foreign antigens, this impaired signalling can occasionally mask responsiveness and yield false negative results (92, 96).

To overcome these limitations, primary human CD8^+^ T-cells are increasingly used as recipients to transduce islet-reactive TCRs (97–100). They present the key advantage to carry the cytotoxic equipment needed for β-cell killing experiments and are transferable to *in vivo* rodent models. In 2015, Babad et al. successfully transferred primary human CD8^+^ T-cells transduced with an IGRP-reactive TCR into immunodeficient HLA-A2-transgenic NSG-A2 mice (97). Although one drawback is the lower (25-50%) TCR transduction efficiency compared to immortalized T-cell lines (97, 98, 101), this can be easily bypassed by sorting the TCR-transduced fraction prior to expansion.

However, as sophisticated as these cellular models can be, co-culturing β-cells with CD8^+^ T-cells remains a reductionist model and does not account for all the complexity of the immune system crosstalk. These models may be fine-tuned by adding other components. For instance, iPS cells could be used to develop isogenic systems by generating other immune cell types such as monocytes, macrophages (102), dendritic cells but also β-cells, all derived from the same donor and co-cultured with TCR-transduced CD8^+^ T-cells in a completely autologous cell system bypassing the problem of alloreactivity (99).

New approaches are currently being developed to derive T-cells from hPSCs, as reviewed in (103). T-cells can be generated from hPSCs either directly or through differentiation to *bona fide* hematopoietic stem cells, which give rise to T lymphocytes and other immune cells. However, the current differentiation protocols need further optimization as the generated T-cells are immature. To allow the maturation of the generated T-cells, others have generated thymic epithelial cells (TECs) from hPSCs, which are important for the positive selection of T-cells. Although the generated TECs were not functional *in vitro*, they were able to differentiate and function when implanted in athymic mice *in vivo* (104). Creating a multi-tissue platform combining SC-islets, SC-T-cells and SC-TECs is a promising avenue, however, considerable challenges need to be addressed before this platform can be used to model T1D *in vitro*.

## β-cell/T-cell interaction readouts

**Figure 6:**
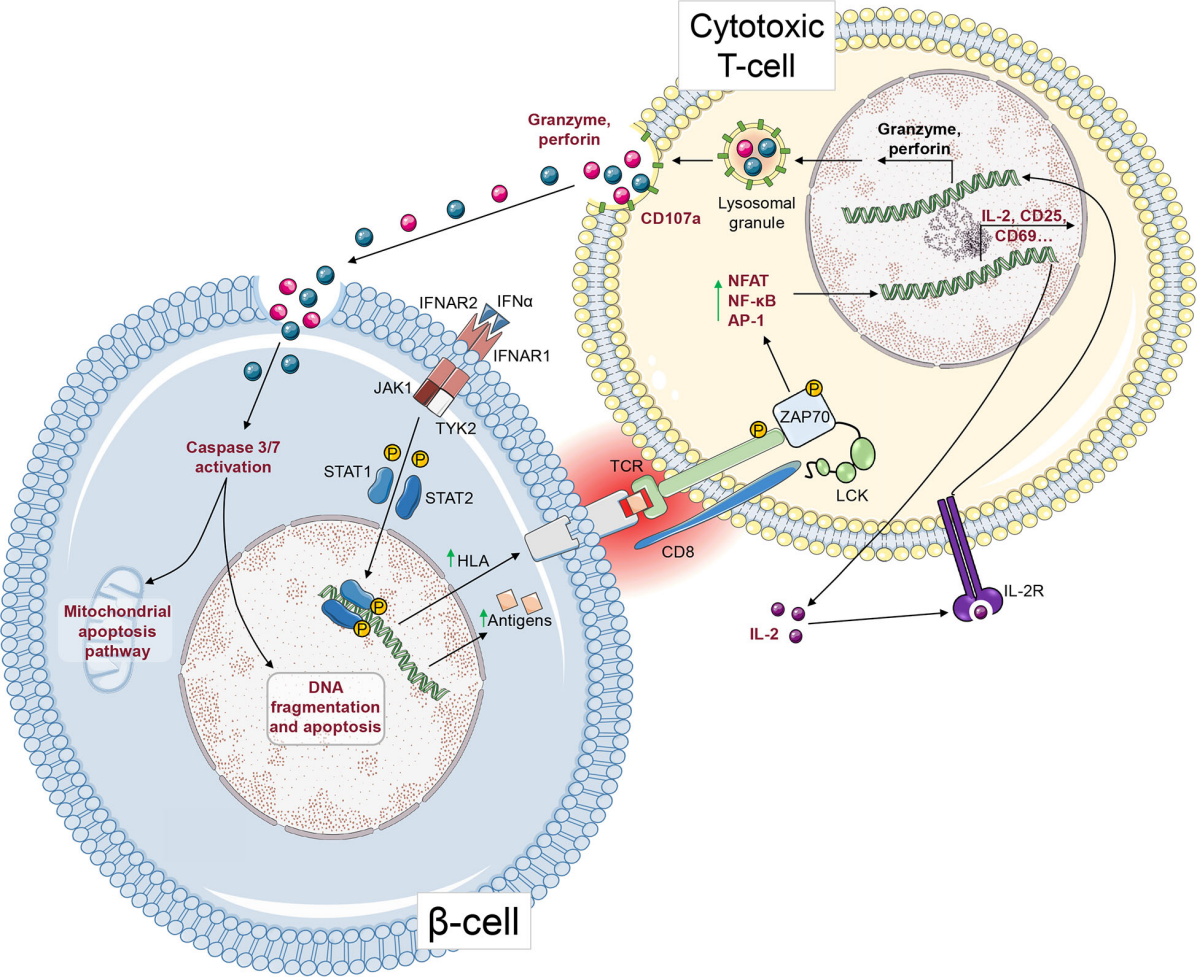
T-cell-mediated cytotoxicity against β-cells. After ligation to its receptor, IFNα induces STAT1 and STAT2 phosphorylation and their nuclear translocation, where they upregulate HLA Class I gene expression. This HLA Class I upregulation leads to increased surface presentation of antigenic peptides that can be recognized by CD8^+^ T-cells *via* their TCR. Upon TCR activation, and after phosphorylation of ZAP70 and other signalling molecules, transcription factors such as NFAT, NF-κB and AP-1 induce the upregulation of genes leading to CD8^+^ T-cell activation. IL-2 secretion acts in an autocrine and paracrine fashion. After activating its receptor, it leads to granzyme and perforin gene upregulation and to the secretion of cytotoxic granules. Granzymes enter the β-cell through pore-forming perforins. Subsequently, granzymes cleave pro-caspase-3 and -7 into active caspases, leading to apoptosis. The steps that can be measured as readouts of β-cell/T-cell crosstalk are depicted in red.

**Figure 7:**
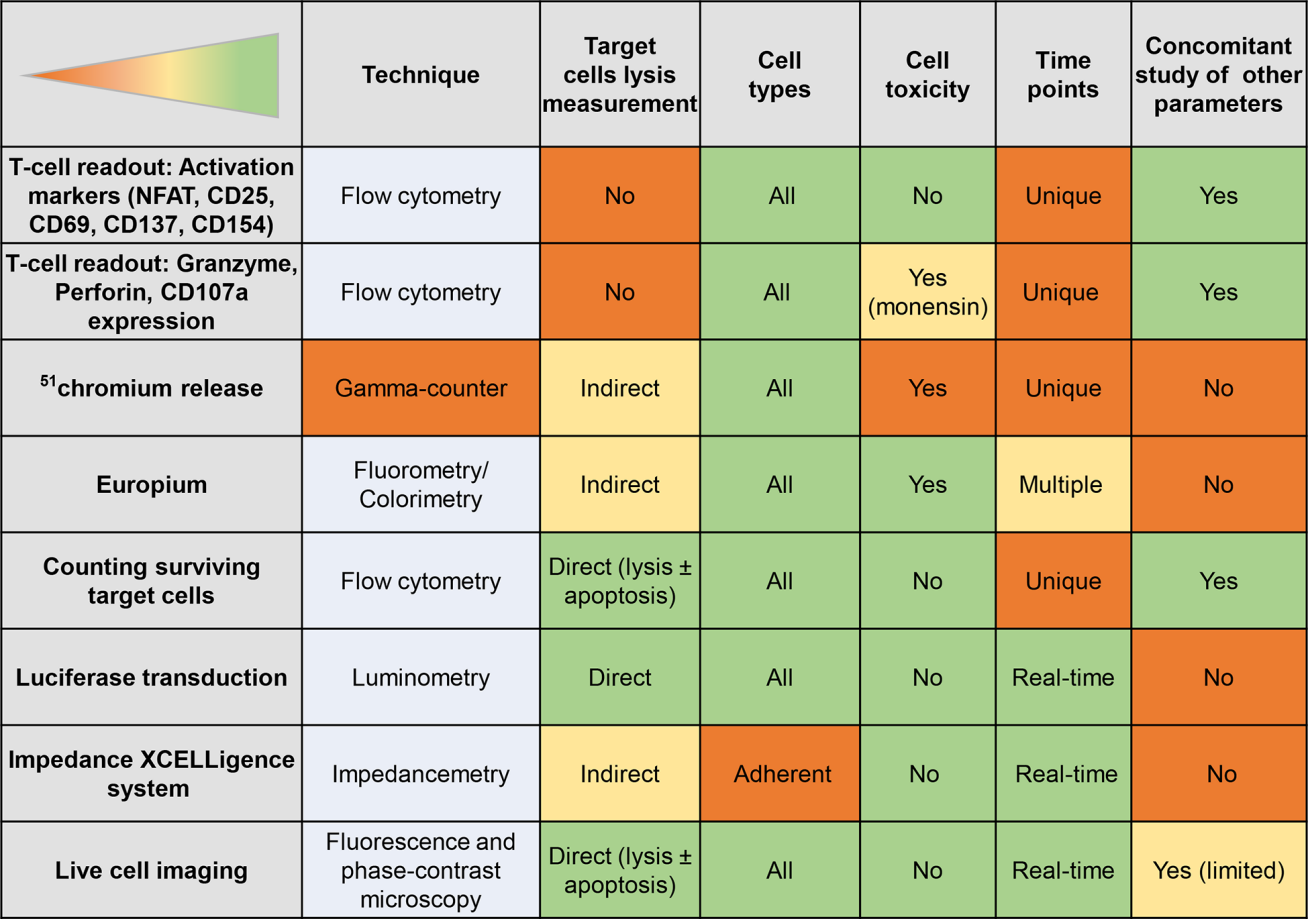
Readouts of T-cell/β-cell crosstalk. The table displays the strengths (green) and weaknesses (orange) of different T-cell or β-cell readouts.

The β-cell and T-cell crosstalk can be studied using readouts that focus on one side of this interaction, alone or in combination. These readouts are schematically depicted in **Figure 6**, with their strengths and weaknesses summarized in **Figure 7**.

### T-cell-based readouts

Some immortalized T-cell lines (e.g. 5KC, Jurkat) are already available with fluorescent reporter gene(s) under the control of NFAT or other promoters (105). These reporter genes can otherwise be introduced by lentiviral transduction (90). After T-cell activation, NFAT is translocated to the nucleus and promotes transcription and translation of cytokine genes, such as IL-2 (106). IL-2 production has therefore been widely used as a T-cell activation marker (107), and the need for IL-2 measurement can be bypassed by reading out the fluorescence of NFAT-driven reporter proteins (e.g. ZsGreen). Surface activation markers such as CD25, CD69, CD137 and CD154 can also be used. However, T-cell activation readouts can only witness the encounter and recognition of the cognate peptide, but they give no proof of cytotoxic activity.

A partial solution to this limitation is provided by readouts of cytotoxic activity, such as the measurement of granzyme release or the surface expression of perforin. All these markers, as well as the release of chemokines/cytokines release such as MIP-1β (the most sensitive readout), IFN-γ, TNF-α or IL-2, can be studied by cytometry or by ELISA and ELISPOT, which can offer complementary approaches to assess CD8^+^ T cells activation (87, 108, 109).

A more convenient readout is however provided by surface CD107a expression. CD107a, also known as lysosomal-associated membrane protein (LAMP)-1, is exposed on the cytoplasmic membrane after its fusion with cytotoxic granules (110). Surface CD107a expression on CD8^+^ T-cells is well correlated to their cytotoxic effect on target cells (110, 111). However, to sensitize CD107a detection, cells must undergo a treatment by protein transport inhibitors such as monensin. Monensin prevents the acidification of endocytic vesicles and avoids the degradation of reinternalized anti-CD107a antibodies (which are typically added throughout the assay to capture the transient surface CD107a expression). However, monensin also inhibits cytokine secretion and more broadly impact T-cell functionality (112). Moreover, an equivalent cytotoxic activity of CD8^+^ T-cells (as measured by surface CD107a expression) may not correspond to an equivalent target cell death if such target cells mount mechanisms modulating cytotoxic vulnerability or protection, as is the case for β-cells.

### β-cell-based readouts

A direct way to analyse T-cell cytotoxicity against β-cells is to measure β-cell lysis upon T-cells encounter.

^51^Chromium release assays have historically been the gold standard readout for killing assays. Target cells are loaded *in vitro* with radioactive ^51^chromium, which is released in the supernatant after target cell death and measured by a gamma counter (113). Nonetheless, this technique requires a dedicated equipment, specific procedures to avoid radioactive hazard and to eliminate radioactive waste. To overcome these disadvantages, some teams developed an alternative technique using non-radioactive chromium read-out by flameless atomic absorption spectroscopy (114).

A similar principle is used in europium release assays. Target cells are labelled with europium, which is released into supernatant upon cell lysis. After chelation by nitrilotriacetic acid, europium can be detected by a fluorometer (115). A more recent strategy to load target cells employs BATDA, which is hydrolysed by intracellular esterases, resulting in accumulation of membrane impermeable TDA inside target cells. Following target cell lysis, TDA is released and chelates europium added to the supernatant (87). This method presents the advantage of being safer and more affordable than ^51^chromium assays, requires lower cell numbers due to higher sensibility and shows comparable results (116, 117). LDH release assays are quite similar in principle to europium assays (97, 118, 119). LDH is a natural enzyme which is released upon cytoplasmic membrane damage. After complexation with NADH and pyruvate, LDH release in supernatant can be estimated by absorbance change using a microplate reader. However, LDH release assay is not suitable for β-cells, as they express very little LDH (120).

Besides these methods relying on the measurement of intracellular tracers released after target lysis, a more direct technique consists in counting the surviving target cells at the end of the assay. To do so, several methods have been developed.

First, target cells can be labelled with fluorescent dyes and their survival can be estimated by flow cytometry, either as relative proportions compared to irrelevant control targets or to calibrations beads, or as absolute counts. The efficiency of this method is comparable to ^51^chromium assays. Moreover, it presents the advantage of allowing the parallel analysis of other markers on both effector and target cells (121–125).

Second, cytotoxic T-cells induce an apoptotic death of target cells. Indeed, granzymes secreted by CD8^+^ T-cells reach the target cytoplasm by pores formed in the plasma membrane by perforin, leading to caspase-3 cleavage and activation (126). Cleaved caspase-3 detection can thus provide a more accurate measurement of the cytotoxic effects on target cells, because this is a process upstream of cell death. This offers a measurement of cytotoxicity on both living and dying cells as compared to the release and killing assays described above that only focus on dead cells. Caspase-3 activation can be detected by flow cytometry by adding a fluorogenic substrate that becomes fluorescent after caspase-mediated cleavage (127). The drawback of this technique is that it requires the use of fresh cells and an immediate flow cytometry acquisition. A more convenient alternative is provided by antibodies directed against cleaved caspase-3 (128, 129). Activated caspase-3 assays have been demonstrated to be more sensitive than ^51^chromium release assays (127–129).

Third, target cells can be transduced to express a luciferase transgene, an ATP-dependent light-emitting enzyme. As dying cells stop emitting after ATP stocks have been consumed, surviving cells can be measured with a luminometer. This method requires transduction of target cells, but offers a real-time follow-up without damaging the cells (130–132).

Another technique presenting the same advantage of a real-time measurement relies on the impedance-based xCELLigence system. Impedance to an electric current running on the bottom of plate wells equipped with electrodes is increased when adherent cells are attached. Dying and detachment of target cells decreases this electrical impedance, which can be continuously measured by the system. However, this technique is limited to adherent cells (3, 133, 134).

Live-cell imaging or real-time microscopy techniques offer novel options for real-time measurement of target cell death. Target cells are labelled with a cytoplasm/nucleus fluorescent dye or transfected with a fluorescent nucleus tracker. Death can be measured in real time by the uptake of a fluorescent dye passively entering the cell as the membrane is damaged and apoptosis can be estimated by a fluorescent caspase-3/7 substrate (135) or by Annexin-V staining. Images are acquired at regular intervals by fluorescence and high-resolution phase-contrast microscopy. In addition to measuring the effects of CD8^+^ T-lymphocytes on target cells, real-time microscopy allows to study their structural interaction. This technique can be used on adherent (135) and non-adherent cells (136), as well as on spheroid organoids (137) such as SC-islets.

## Applications of *in vitro* β-cell killing platforms

New systems are emerging to study the autoimmune interactions between human β-cells and immune cells. This is facilitated by the recent development of human β-cell lines and the possibility to generate SC-islets and TCR-transduced primary T-cells from healthy and T1D subjects. The choice of the model depends on the parameters under study, the chosen readout and the affordability of the platform. While SC-islets provide high β-cell functionality and easy genetic engineering, their 3D cluster morphology can make some readouts (such as measuring surviving target cell numbers) more challenging, making β-cell lines more convenient for some experiments.

The role of genetic variants involved in β-cell/T-cell crosstalk can be investigated using genetically engineered SC-islets to elucidate the vulnerability or protection potential of the genetic modification under study. Another challenge that can be tackled using β-cell/T-cell models is how to hide β-cells from immune surveillance to avoid autoimmune or allogenic immune attack for β-cell replacement therapies. This is relevant also for autologous iPSC derivatives, since graft (auto)immune rejection also occurs in this fully HLA-matched setting (138, 139).

Human β-cells have the ability to upregulate the expression of multiple factors to interact with the immune system in response to inflammatory environment. HLA class I upregulation is a major driver of T1D pathogenesis (27, 140), as these molecules present self-antigens to autoreactive CD8^+^ T-cells. To counteract the inflammatory response, β-cells upregulate the Programmed Death-Ligand 1 (PD-L1) which acts as an immune checkpoint inhibitor by binding to its receptor PD-1 on T-cells, thus inhibiting TCR signalling and T-cell activation (141, 142). It is considered an important protective immunomodulatory factor when expressed in β-cells in response to pro-inflammatory cytokines. This protection mechanism is similar to that mounted by cancer cells, which upregulate PD-L1 to evade immune surveillance (143). This provided the basis of blocking anti-PD-1/PD-L1 antibody therapeutics (144), with agonist anti-PD-1 antibodies under development to achieve an opposite tolerogenic effect for autoimmune diseases. β-cell/T-cell crosstalk models may provide valuable platforms to screen for such therapeutics. They may also elucidate the dynamics of upregulation and interaction between PD-1/PD-L1 and other immunomodulatory receptor/ligand pairs. A similar application is envisaged for candidate β-cell-protective agents, e.g. verapamil (145), JAK, STAT and TYK2 inhibitors, as exemplified in our proof-of-concept study (74). Using a TYK2 inhibitor and an *in vitro* β-cell/T-cell model, we observed only a partial protection from β-cell destruction, due to an inhibition of the IFNα-induced upregulation on both HLA Class I and PD-L1.

Although islet-reactive T-cells exist in healthy individuals, they are not residing in islets, as is the case for T1D patients (3, 27, 146). Interestingly, T-cells cultured with autologous SC-islets from non-diabetic and T1D donors showed neither activation nor β-cell target cytotoxicity unless SC-islets were preliminarily exposed to endoplasmic reticulum stressors (147). This emphasizes the increasing appreciation of the active role that β-cells play in shaping their autoimmune vulnerability (148–151), which will be another paramount aspect to address using β-cell/T-cell crosstalk models.

Similar to primary human islets, SC-islets have the ability to upregulate HLA Class I molecules and PD-L1 upon pro-inflammatory cytokine exposure (152, 153). They can also present epitopes such as preproinsulin (PPI) peptides to CD8^+^ cytotoxic T-cells (154). Disruption of the β2-microglobulin (*B2M*) gene diminishes surface HLA class I expression, thus preventing CD8^+^ T-cell-mediated cytotoxicity. However, since non-classical HLA class I molecules HLA-E and HLA-G inhibit the activation of the innate immune natural killer (NK) cells, *B2M* knockout cells display increased vulnerability to NK-mediated lysis (155). Different approaches are being exploited to create hypoimmunogenic stem cells that can avoid both T-cells and NK-cells. While overexpressing HLA-E fused to B2M reduced NKG2A^+^ NK-cells-mediated lysis of *B2M* knockout cells (156), it was not adequate to suppress KIR2DL1–4^+^ NK-cell populations, which are suppressed by HLA-C or HLA-G (157, 158). Indeed, another approach disrupting HLA-A and HLA-B, but not HLA-C, improved NK- cell evasion (159). Other studies used a multiplexed CRISPR/Cas9 system to delete HLA-A/-B/-C genes and HLA class II genes by targeting *CIITA.* In addition to these deletions, overexpressing the immunomodulatory factor CD47, alone (160) or together with PD-L1 and HLA-G (161), inhibited the activation of T-cells, NK-cells, and macrophages. Although upregulating immunomodulatory factors in β-cells enhanced their survival against immune cells, introducing overexpression cassettes in stem cells is prone to silencing in their differentiated progeny (162–164). Therefore, an alternative approach is to knockout the NK-activating ligands expressed in SC-islets. Profiling these ligands showed high gene expression levels of *B7-H3* and *CD155* throughout SC-islets differentiation. Generating a triple knockout of *B2M*, *B7-H3* and *CD155* demonstrated a significant reduction in T-cell- and NK-cell-mediated lysis *in vitro* and *in vivo* (165). However, evaluating long-term survival and functionality requires further assessment.

These examples of double-edged genetic manipulation exemplify the need for more complex *in vitro* models recapitulating the action of different immune cell mediators. It will also be important to test the possibility of restoring immune surveillance in the case of β-cell infection or malignant transformation, or whether harnessing these transplanted β-cells with kill-switch suicide genes may trigger autoimmunity by releasing self-antigens. In this perspective, the evolution of these *in vitro* platforms toward *in vivo* systems using humanized mouse models is highly desirable to thoroughly address these concerns.

## Conclusions and perspectives

Development of human β-cell lines and of genetically engineered SC-islets from T1D or healthy donors combined with T-cell co-culturing platforms allows a quantitative measurement of β-cell killing using different readouts. Recent technological advancements in developing these assays open new venues to model a complex disease like T1D. Investigating the interaction between β-cells and cytotoxic immune cells in autologous or allogeneic settings will shed light on the pathogenic mechanisms and help identifying novel therapeutic targets for T1D and β-cell replacement strategies. Genetic and drug screening using these assays may identify new targets to decrease β-cell vulnerability and/or T-cell aggressiveness and to allow transplanted β-cells to escape immune recognition.

## Author contributions

CH and HI contributed equally to writing the review and share first authorship. TO acquired funding and participated in manuscript writing. RM conceived and supervised the review, acquired funding and participated in manuscript writing. All authors contributed to the article and approved the submitted version.
